# RETRACTED: Kalicińska et al. Pneumonia in Patients with Chronic Lymphocytic Leukemia Treated with Venetoclax-Based Regimens: A Real-World Analysis of the Polish Adult Leukemia Group (PALG). *Cancers* 2024, *16*, 4168

**DOI:** 10.3390/cancers17122032

**Published:** 2025-06-18

**Authors:** Elżbieta Kalicińska, Paula Jabłonowska-Babij, Marta Morawska, Elżbieta Iskierka-Jażdżewska, Joanna Drozd-Sokołowska, Ewa Paszkiewicz-Kozik, Łukasz Szukalski, Judyta Strzała, Urszula Gosik, Jakub Dębski, Iga Andrasiak, Anna Skotny, Krzysztof Jamroziak, Tomasz Wróbel

**Affiliations:** 1 Clinical Department of Hematology, Cell Therapies and Internal Diseases, Wroclaw Medical University, 50-367 Wroclaw, Poland; pjablonowska@usk.wroc.pl (P.J.-B.); tomasz.wrobel@umw.edu.pl (T.W.); 2 Experimental Hematooncology Department, Medical University of Lublin, 20-059 Lublin, Poland; mmorawska79@gmail.com; 3 Department of General Hematology, Copernicus Memorial Hospital, Medical University of Lodz, 90-419 Lodz, Poland; elzbieta.iskierka-jazdzewska@umed.lodz.pl; 4 Department of Hematology, Transplantation and Internal Medicine, Medical University of Warsaw, 02-091 Warsaw, Poland; johna.dr@poczta.fm (J.D.-S.); k.m.jamroziak@gmail.com (K.J.); 5 Department of Lymphoid Malignancies, National Research Institute of Oncology, 02-781 Warsaw, Poland; ewa.paszkiewicz-kozik@pib-nio.pl; 6 Department of Hematology, Collegium Medicum in Bydgoszcz, Nicolaus Copernicus University in Toruń, 85-168 Toruń, Poland; lukaszszukalski@gmail.com; 7 Department of Hematology and Bone Marrow Transplantation, Pomeranian Hospitals in Gdynia, 81-519 Gdynia, Poland; judytastrzala87@gmail.com; 8 Department of Hematology, St. John’s Cancer Center in Lublin, 20-090 Lublin, Poland; u.gosik@gmail.com; 9 Department of Hematology, Provincial Specialist Hospital in Legnica, 59-220 Legnica, Poland; jmdebski@gmail.com; 10 Individual Business Activity, ul. Zielna 28b/3, 51-313 Wroclaw, Poland; igaandrasiak@gmail.com; 11 Harvard T.H. Chan School of Public Health—Executive and Continuing Education, Harvard University, Boston, MA 02115, USA; annaskotny@gmail.com

The *Cancers* journal retracts the article titled “Pneumonia in Patients with Chronic Lymphocytic Leukemia Treated with Venetoclax-Based Regimens: A Real-World Analysis of the Polish Adult Leukemia Group (PALG)” [1], cited above.

Following publication, concerns were brought to the attention of the Editorial Office regarding an overlap between this article [1] and another article [2], which had been published by a different publisher prior to the submission and publication of [1].

There was a misunderstanding during the editorial processing of article [2], which led to the publication of the paper. The authors had initially intended to withdraw article [2] after its publication in early view, and had received confirmation from the eJHaem Editorial Office that this could be done. Based on this understanding, article [1], authored by the same group, was subsequently submitted and published. However, it was later clarified that the withdrawal of article [2] was not feasible, as it had already been assigned a digital object identifier (DOI) and published, which made its withdrawal impossible according to COPE guidelines.

Adhering to our standard procedure, an investigation was conducted by the Editorial Office and the Editorial Board, which confirmed a significant overlap between the two publications.

Given the level of overlap and the respective publication dates, the Editorial Board considers this publication [1] to be redundant and have decided to retract this article as per MDPI’s retraction policy (https://www.mdpi.com/ethics#_bookmark30) and in line with the Committee on Publication Ethics’s retraction guidelines (https://publicationethics.org/retraction-guidelines).

This retraction was approved by the Editor-in-Chief of the journal *Cancers*. The authors agreed to this retraction.
